# Economic evaluation of multidisciplinary rehabilitation treatment versus cognitive behavioural therapy for patients with chronic fatigue syndrome: A randomized controlled trial

**DOI:** 10.1371/journal.pone.0177260

**Published:** 2017-06-02

**Authors:** Desirée Vos-Vromans, Silvia Evers, Ivan Huijnen, Albère Köke, Minou Hitters, Nieke Rijnders, Menno Pont, André Knottnerus, Rob Smeets

**Affiliations:** 1 Revant Rehabilitation Centres, Breda, The Netherlands; 2 Department of Health Services Research, Research School CAPHRI Maastricht University, Maastricht, The Netherlands; 3 Trimbos Institute, National Institute of Mental Health and Addiction, Utrecht, The Netherlands; 4 Department of Rehabilitation Medicine, Research School CAPHRI Maastricht University, Maastricht, The Netherlands; 5 Department of Rehabilitation Medicine, Academic Hospital Maastricht, Maastricht, The Netherlands; 6 Adelante Centre of Expertise in Rehabilitation and Audiology, Hoensbroek, The Netherlands; 7 Libra Rehabilitation and Audiology, Eindhoven, The Netherlands; 8 Reade Centre of Rheumatology and Rehabilitation, Amsterdam, The Netherlands; 9 Department of General Practice, Research School CAPHRI Maastricht University, Maastricht, The Netherlands; TNO, NETHERLANDS

## Abstract

**Background:**

A multi-centre RCT has shown that multidisciplinary rehabilitation treatment (MRT) is more effective in reducing fatigue over the long-term in comparison with cognitive behavioural therapy (CBT) for patients with chronic fatigue syndrome (CFS), but evidence on its cost-effectiveness is lacking.

**Aim:**

To compare the cost-effectiveness of MRT versus CBT for patients with CFS from a societal perspective.

**Methods:**

A multi-centre randomized controlled trial comparing MRT with CBT was conducted among 122 patients with CFS diagnosed using the 1994 criteria of the Centers for Disease Control and Prevention and aged between 18 and 60 years. The societal costs (healthcare costs, patient and family costs, and costs for loss of productivity), fatigue severity, quality of life, quality-adjusted life-year (QALY), and cost-effectiveness ratios (ICERs) were measured over a follow-up period of one year. The main outcome of the cost-effectiveness analysis was fatigue measured by the Checklist Individual Strength (CIS). The main outcome of the cost-utility analysis was the QALY based on the EuroQol-5D-3L utilities. Sensitivity analyses were performed, and uncertainty was calculated using the cost-effectiveness acceptability curves and cost-effectiveness planes.

**Results:**

The data of 109 patients (57 MRT and 52 CBT) were analyzed. MRT was significantly more effective in reducing fatigue at 52 weeks. The mean difference in QALY between the treatments was not significant (0.09, 95% CI: -0.02 to 0.19). The total societal costs were significantly higher for patients allocated to MRT (a difference of €5,389, 95% CI: 2,488 to 8,091). MRT has a high probability of being the most cost effective, using fatigue as the primary outcome. The ICER is €856 per unit of the CIS fatigue subscale. The results of the cost-utility analysis, using the QALY, indicate that the CBT had a higher likelihood of being more cost-effective.

**Conclusions:**

The probability of being more cost-effective is higher for MRT when using fatigue as primary outcome variable. Using QALY as the primary outcome, CBT has the highest probability of being more cost-effective.

**Trial registration:**

ISRCTN77567702.

## Introduction

Chronic fatigue syndrome (CFS) is defined as medically unexplained disabling fatigue that persists for more than six months, often leading to decreased quality of life, restrictions in personal and social activities and a limited ability to work [1,2]. The prevalence of CFS is estimated between 0.2–2.6% worldwide [3]. However, the burden of CFS to society has been measured only in a few countries. In the United States, the annual direct total costs per patient were estimated to be between $2,342 and $8,675 depending on the sample used [4,5]. Annual total losses of productivity per patient in the US were estimated at $20,000 [6]. In another study, based on a sample in the United Kingdom, annual productivity losses per patient were estimated at £22,684 [7]. In the Netherlands annual costs for the healthcare of patients with unexplained physical symptoms, including CFS, were estimated at €3,123 per patient [8]. These costs, together with annual work-related costs and paid substitution for doing domestic tasks were estimated at €6,815 per patient [8]. Although this study involved patients with a variety of unexplained physical symptoms, it provides an indication of the high economic burden and the need to investigate new treatments and their benefits for the patient and society. One commonly used treatment with evidence supporting its effectiveness and cost-effectiveness is cognitive behavioural therapy (CBT) [9]. Previous studies showed that CBT is a cost-effective treatment when compared with guided support groups [10,11], specialist medical care or adaptive pacing therapy [10,12]. QALY is a commonly used summary measure of health-related quality of life, taking account of both quality and quantity of life. One QALY equates to living one year in perfect health. When fatigue is the primary outcome, CBT is equally cost-effective as counseling [13] and more cost-effective in comparison with guided support groups or a natural course group [11]. To further improve effectiveness, it has been advocated to investigate multidisciplinary treatments that include CBT in combination with other interventions [9]. In response to this recommendation a multidisciplinary rehabilitation treatment (MRT) was developed and studied in a randomized controlled trial (RCT), comparing MRT with CBT [14]. The results of that trial revealed that one year after start of treatment, MRT was more effective in reducing the severity of fatigue, in comparison with CBT (*P* = 0.02) [14]. No statistically significant differences in quality of life were found between MRT and CBT. Studies investigating the cost-effectiveness of MRT for patients with CFS are scarce. The only study investigating the cost-effectiveness of a rehabilitation treatment is the Fatigue Intervention by Nurses Evaluation (FINE) trial [15]. This trial compared a rehabilitation treatment with supportive listening and treatment as usual. The rehabilitation treatment in the FINE trial was a mono-disciplinary treatment with other treatment modalities as provided in MRT. Results of the FINE trial indicated that pragmatic rehabilitation was not cost-effective when looking at the costs per QALY measured by the EuroQol-5D-3L (EQ-5D-3L). The rehabilitation treatment studied in the FINE trial was different and not comparable to MRT, making it difficult to generalize the results of the FINE trial to all rehabilitation treatments. The need to study the cost-effectiveness remained. Therefor as an integral part of the RCT, the cost-effectiveness was analyzed. The aim of the present study is to report the cost-effectiveness and cost-utility from a societal perspective comparing MRT and CBT. For the cost-utility the QALY, generated from the standard Dutch version of the EQ-5D-L, was used. The EQ-5D-3L, which is a generic measure, assesses five domains of the quality of life, with only three scoring levels. Recently, in studies of patients with chronic illnesses in which fatigue is a common complaint, the use of disease-specific outcome measures in addition to generic measures is advised, since the generic measures might not be sensitive enough to measure change after treatment [16–18]. To provide a more disease-specific outcome, the fatigue severity measured by the Checklist Individual Strength (CIS) [19], subscale fatigue and quality of life measured with the Short-Form 36 (SF-36) [20] were included. Studies regarding the cost-effectiveness of MRT or MRT in comparison with CBT have never been done. The aim of the present study is to report the cost-effectiveness and cost-utility from a societal perspective comparing MRT and CBT in terms of reduction in fatigue and gain in health-related quality of life and gains in QALYs over one year. It is hypothesised that MRT is more cost-effective and shows a higher cost-utility compared with CBT [14].

## Methods

### Study design and participants

This multi-centre, two-arm RCT was registered in the ISRCTN database (Trial Registration Number: ISRCTN77567702). A detailed description of the design of the trial has been published elsewhere [21]. The S1 File represents the design of the trial. Patients referred to four rehabilitation centres (secondary healthcare) in the Netherlands: the Revant Rehabilitation Centres (location Breda), Libra Rehabilitation and Audiology in Eindhoven, Reade Centre for Rheumatology and Rehabilitation in Amsterdam and Adelante Rehabilitation Centre in Hoensbroek between December 2008 and January 2011 were invited to participate if they met the US Centers for Disease Control and Prevention (CDC-94) criteria for CFS [1]. Other inclusion criteria were: a CIS fatigue subscale score of 40 or more [22,23], willingness to participate in a treatment aimed at changing behaviour, age between 18 and 60 years, and comprehension of written and verbal Dutch. Patients were excluded if they suffered from a medical condition explaining the presence of chronic fatigue, had a psychiatric or depressive disorder, dementia, anorexia, bulimia nervosa, alcohol and/or drug abuse, a body mass index of 45 or more, or were pregnant. Patients who had already received CBT or MRT for CFS in the past, or had to travel more than one hour to the nearest participating rehabilitation centre, were also excluded. Outcomes were assessed at baseline, 4, 14, 26 weeks (directly after end of treatment) and 52 weeks after start of treatment. After baseline, patients were randomized to CBT or MRT. An economic evaluation study consisting of a cost-effectiveness analysis (primary outcome: fatigue) and a cost-utility analysis (primary outcome: QALY) was embedded in the RCT. All patients provided written informed consent. The Research Ethics Committee of Rotterdam, TWOR (reference 2008/22) approved the study on 27 November 2008. The CONSORT and CHEERS checklists of the trial are listed in S2 and S3 Files.

### Interventions

In MRT, a consultant in rehabilitation medicine, social worker, psychologist, physical therapist and occupational therapist worked as an interdisciplinary team together with the patient for 6 months. The protocol prescribed a total of 44.5 face-to-face contact hours. Gradual reactivation, pacing, mindfulness, body awareness therapy, normalising sleep-wake rhythm and social reintegration were combined with CBT and tailored to the individual needs and goals of the patient.

CBT is a psychotherapeutic approach in which elements of behavioural and cognitive therapy approaches are incorporated to change behavioural and cognitive factors, which are assumed to perpetuate the symptoms of CFS [24]. CBT was delivered by a psychologist or cognitive behavioural therapist. The protocol of CBT prescribed a total of 16 face-to-face contact hours during 6 months. Both treatments aimed at decreasing the severity of fatigue and increasing quality of life. Treatments have been described earlier [14].

### Outcome measures

#### Effects

For the cost-effectiveness analysis, the primary outcome is fatigue severity, which was measured by the CIS fatigue subscale (CIS fatigue; score ranging from 8–56, lower scores indicate less fatigue) [19]. In the cost-effectiveness analyses the CIS fatigue scores were recoded: a higher score indicates a more positive effect (less fatigue). In using the CIS fatigue subscale, there are different methods for defining improvement. One is to change the CIS score to a dichotomous variable of improvement (CIS improvement). A score lower than 35 on the CIS fatigue subscale was labelled as improved [25]. A higher score was labelled as not improved.

For the utility analysis, QALYs were generated from the standard Dutch version of the EQ-5D-3L [26]. The EQ-5D-3L contains five dimensions of health-related quality of life: mobility, self-care, daily activities, pain/discomfort and depression/anxiety. Each dimension can be rated on one of three levels: no problem, some problems and major problems. The five dimensions were aggregated into a health state. Utility values were calculated for these health states, using preferences elicited from a general population from the UK, the so-called Dolan algorithm [27]. Utilities were calculated for every assessment. The accrual of QALYs from baseline to the 52 week follow-up was calculated using the area under the curve, assuming a linear change between each available time point.

As a secondary outcome for the cost-effectiveness and cost-utility analyses, health-related quality of life was measured by the SF-36 [20]. The SF-36 has 8 subscales which were combined in two summary scores; the mental (MCS) score and the physical component summary score (PCS). The outcomes at the 52 week follow-up were used in the cost-effectiveness analysis. To measure the opinion of the patient regarding his/her improvement, the patient was asked to fill in the Improvement and satisfaction questionnaire (EET). Question 4 of the EET: “Is there a difference in your daily activities now compared to your situation before treatment started”, was used in the sensitivity analysis to give an indication of the extent to which a patient feels he or she has improved [14].

#### Costs

Costs from a societal perspective were registered from baseline until 52 weeks after baseline. These costs included healthcare costs, patient and family costs, and costs coming from productivity losses. Healthcare costs included costs for all healthcare services including costs for the interventions and costs for all medication (including over the counter medication). Patient and family costs included travelling costs to all healthcare institutions, parking costs for the visits to the hospital and domestic help which would be normally done by the patient her/himself and unpaid informal care provided by family or other unpaid people. Productivity losses were measured by costs of absenteeism of work. Table 1 shows the categories identified. The Trimbos/iMTA questionnaire for Costs associated with Psychiatric Illness (TiC-P) was used to measure the costs of healthcare, patient and family and productivity losses [28]. TiC-P, part I, a questionnaire on absence from work, informal care and domestic help, was filled in every month. TiC-P, part II, a questionnaire on healthcare costs was filled in every three months.

**Table 1:** Costs and valuation per category.

Category	Unit	Valuation (in €)
**Healthcare costs**		
General practitioner	Per consult	29.74
Regional Institution for ambulatory mental healthcare (RIAGG)	Per consult	115.91
Psychiatrist, psychologist, psychotherapist in private practice	Per consult	95.60
Psychiatrist, psychologist in outpatient academic hospital	Per consult	36.12
Psychiatrist, psychologist in outpatient in general hospital	Per consult	30.80
Psychiatrist, psychologist in outpatient psychotherapeutic setting or in psychiatric hospital	Per consult	183.76
Psychiatrist, psychologist in outpatient in other hospital	Per consult	108.61
Company physician	Per consult	173.90
Medical specialist (outpatient hospital)	Per consult	33.46
Paramedics	Per consult	32.50
Social worker	Per consult	69.04
Centre for alcohol and drug abuse (CAD)	Per consult	98.28
Alternative healer	Per consult	59.31
Self-help group	Per consult	67.76
Daytreatment in academic hospital	Per day	252.81
Daytreatment in general hospital	Per day	168.89
Daytreatment in psychotherapeutic setting or psychiatric hospital	Per day	163.58
Daytreatment in other setting	Per day	187.22
Admission in academic hospital	Per day	610.77
Admission in general hospital	Per day	462.06
Admission in psychotherapeutic setting or psychiatric hospital	Per day	246.43
Admission in other setting	Per day	391.42
Medication prescribed by the general practitioner or medical specialist including delivery costs (€6.28).	Per piece	^a^
Costs for writing a prescription by the general practitioner or medical specialist	Per prescription	14.87
Costs for home care by a trained professional	Per hour	37.62
**Patient and family costs**		
Travelling costs for the interventions, external interventions and for retrieving medication at the pharmacy^b^	Per km	0.21
Parking costs for visiting the hospital	Per visit	3.19
Domestic help or unpaid informal care from family/friends	Per hour	13.23
**Productivity losses**		
Hours of absenteeism	Per hour	Hourly wage x 0.8

^a^ Costs for medication were calculated by taking the average of the highest and lowest price per piece of medicine in 2012 on www.medicijnkosten.nl.

^b^ Travelling costs are counted for every visit and once every 3 months for retrieving medication at the pharmacy. In the Dutch manual for cost analysis in healthcare research the distance is given to the nearest institution in 2008, namely the general practitioner 1.1 km, RIAGG 7.0 km, psychiatrist, psychologist, psychotherapist in private practice, social worker and alternative healer 3.6 km, hospital, company physician, self-help group and CAD 7.0 km, paramedics 2.2 km, rehabilitation centre 26.4 km.

The valuation of healthcare costs and the patient and family costs was based on the updated Dutch manual for cost analysis in healthcare research [29,30]. Costs were indexed to the year 2012 by means of the consumer price indexes of the Dutch Central Bureau of Statistics. For care for which no costs-guidelines were available, estimations of the costs were made based on the real costs or on population-based estimates from literature. Table 1 shows the costs per identified category. Medication costs were based on the tariffs from the Dutch College of Health Insurance (www.medicijnkosten.nl). Treatment hours for MRT and CBT were registered by the therapist and the consultant in rehabilitation medicine after each treatment session. The duration of the treatment sessions was added up and costs calculated using the Dutch diagnosis-dependent treatment combination for cost-pricing the interventions (www.dbconderhoud.nl). Following this procedure, the following costs per treatment category were used: 0–2 hours of outpatient rehabilitation treatment €200, 2–6 hours of treatment €539, 6–18 hours €1,364, 18–49 hours €3,557, 49–129 hours €8,620, 129–299 hours €19,392, 299 hours and more €37,268.

To value the travelling costs, the number of visits to the healthcare services was multiplied by the mean distance and then multiplied by the costs per kilometre [29]. An assumption of 26.4 kilometres was made for the mean distance to a rehabilitation centre. This assumption is based on the ratio between number of hospitals divided by the number of rehabilitation centres in the Netherlands, multiplied by the mean distance between a hospital and a patient’s home.

Every month the patient reported the days lost from work due to fatigue as well as his/her wages (TiC-P, part II). Following the human capital approach, the total hours of absenteeism was multiplied by the hourly wages and afterwards multiplied by a factor of 0.8. The 0.8 factor is a correction because productivity in the Netherlands decreases by a factor of 0.8 as working hours decrease due to absenteeism [29]. The national mean age and gender-specific wages were used when the patient preferred not to fill in his/her wages.

### Statistical methods

An intention-to-treat analysis was used, which means the data of all patients initially assigned to a treatment were analysed, regardless of whether or not they completed or received the treatment. Patients were included in the cost-effectiveness analysis if they filled in 75% of all 16 TiC-P questionnaires: otherwise they were excluded from the analyses. Any remaining missing values in the TiC-P questionnaires were imputed by using the last observation carried forward. If variables from the previous time period were missing, the last observation carried backward was used. If variables were missing in every monthly or 3-monthly questionnaire of a participant, the averages of the analysed participants were used.

#### Effectiveness analysis

Baseline differences between CBT and MRT of the primary and secondary outcomes were calculated using t-tests. The longitudinal effect of MRT versus CBT on the outcomes was assessed using linear mixed models, fit using restricted maximum likelihood [14]. Along with treatment allocation, time (in weeks from baseline), and interaction of time by treatment allocation, rehabilitation centre was included as a fixed factor, because randomization was stratified by centre. Choice of the best-fitting covariance structure, i.e. structure of variances over different time-points and correlations between time-points, was based on Akaike’s Information Criterion. Two-sided *P*-values smaller than or equal to 0.05 were considered statistically significant. No missing outcome data were imputed for these linear mixed models, instead the likelihood approach was used. Analyses were performed using SPSS Statistics for Windows (version 20.0; IBM Corp., Armonk, NY, USA.).

#### Cost-effectiveness analysis

The costs from a societal perspective during the follow-up period of 52 weeks were cumulated and the total costs from the two intervention groups were compared by the non-parametric bootstrapping method with 95% confidence intervals in percentiles [31]. Base-case cost-effectiveness and cost-utility analyses and sensitivity analyses were done. In the base-case analysis, the cost-effectiveness was performed by relating the mean total costs to the severity of fatigue and quality of life at 52 weeks. Severity of fatigue and quality of life are adjusted for baseline values. Costs are the total costs from baseline until 52 weeks follow-up. A cost-utility analysis was performed by relating the mean total costs to the mean health utility (QALY) scores of both groups. The costs per QALY of both treatments were compared. The incremental cost-effectiveness ratio (ICER) was determined on the basis of incremental costs and the effects of the MRT in comparison with CBT. The cost-effectiveness ratio presented the costs per unit of outcome and the cost-utility ratio focused on the incremental cost per QALY gained. The robustness of the ICER was checked by non-parametric bootstrapping to quantify the uncertainty around the ICER. The bootstrapped cost-effectiveness ratios were subsequently plotted in a cost-effectiveness plane. The choice of treatment depends on the maximum amount of money society is prepared to pay for a gain in effectiveness, which is called the ceiling ratio. In the Netherlands, no explicit ceiling ratio or ICER threshold value is defined, but the Council of Public Health and Health Care advised using a ceiling ratio for the QALY related to the burden of disease [32]. The burden of disease ranges from ‘0’, indicating no burden of disease, to a score of ‘1’, indicating a maximum burden of disease. Although the exact burden of disease for patients with CFS is unknown, NICE guidelines for the treatment of CFS declare the burden of disease to be comparable with other chronic conditions such as multiple sclerosis and rheumatoid arthritis [33]. A more recent study [8] showed that the burden of disease among patients with unexplained physical symptoms, including CFS, is high and comparable with major depression and cancer. A report issued in 1998 from the National Institute for Public Health and the Environment (RIVM) [34] describes the burden of disease for different illnesses: for example, the burden of disease for multiple sclerosis is between 0.33 and 0.67, which is comparable with moderate to severe depression (burden of disease 0.35–0.76). Since the exact burden of disease of CFS is unknown, the burden is estimated to be between 0.33 and 0.76. Following the report “Zinnige en duurzame zorg” (“Sensible and sustainable care”) from the Council of Public Health and Health Care [32], the estimated willingness to pay for treatment for patients with CFS lies between €27,000 to €60,000 for one QALY. The bootstrapped ICERs were depicted in cost-effectiveness and cost-utility acceptability curves showing the probability that MRT is cost-effective while using a range of ceiling ratios. Costs, cost-effectiveness, cost-utility and sensitivity analyses were carried out using Microsoft Office Excel 2003.

#### Sensitivity analysis

Finally, different sensitivity analyses were performed to demonstrate the robustness of our base-case findings. Sensitivity analyses were performed by varying different parameters on which assumptions were made. The following sensitivity analyses were performed: (1a-b) Costs were calculated using the friction cost method instead of the human capital approach and (2a-b) costs were calculated from a healthcare perspective instead of a societal perspective. During the base-case analysis it was noticed that as patients had to fill in their hours of absence while reintegrating or working part-time due to their illness, inconsistencies were found within the answers. Assumptions had to be made in order to calculate the correct hours of absence. The estimated minimum hours of absence was used in the base-case analysis. The estimated maximum hours of absence was used in sensitivity analysis (3a-b). To analyse whether results of the base case analyses regarding the disease-specific outcome were the same using another outcome, sensitivity analyses 4 and 5 were performed. In sensitivity analysis 4, an overall improvement score was used. Improvement was measured by the Improvement and satisfaction questionnaire (EET), question 4 “Is there a difference in your daily activities now compared to your situation before treatment started? (‘1’ = improved and ‘0’ = not improved) [14]. (5) In the base case the incremental costs per unit of outcome on the CIS fatigue subscale were calculated. Since a 1-point improvement is not considered to be a clinically significant improvement, a sensitivity analysis was performed in which the improvement was calculated using the CIS fatigue subscale as dichotomous variable of improvement. Previous studies [23,25] used the CIS cut-off score of 35 to measure clinically significant improvement [35]. Finally, a sensitivity analysis (6) was performed using the Dutch algorithm instead of the UK algorithm to calculate the QALY.

## Results

**Fig 1:**
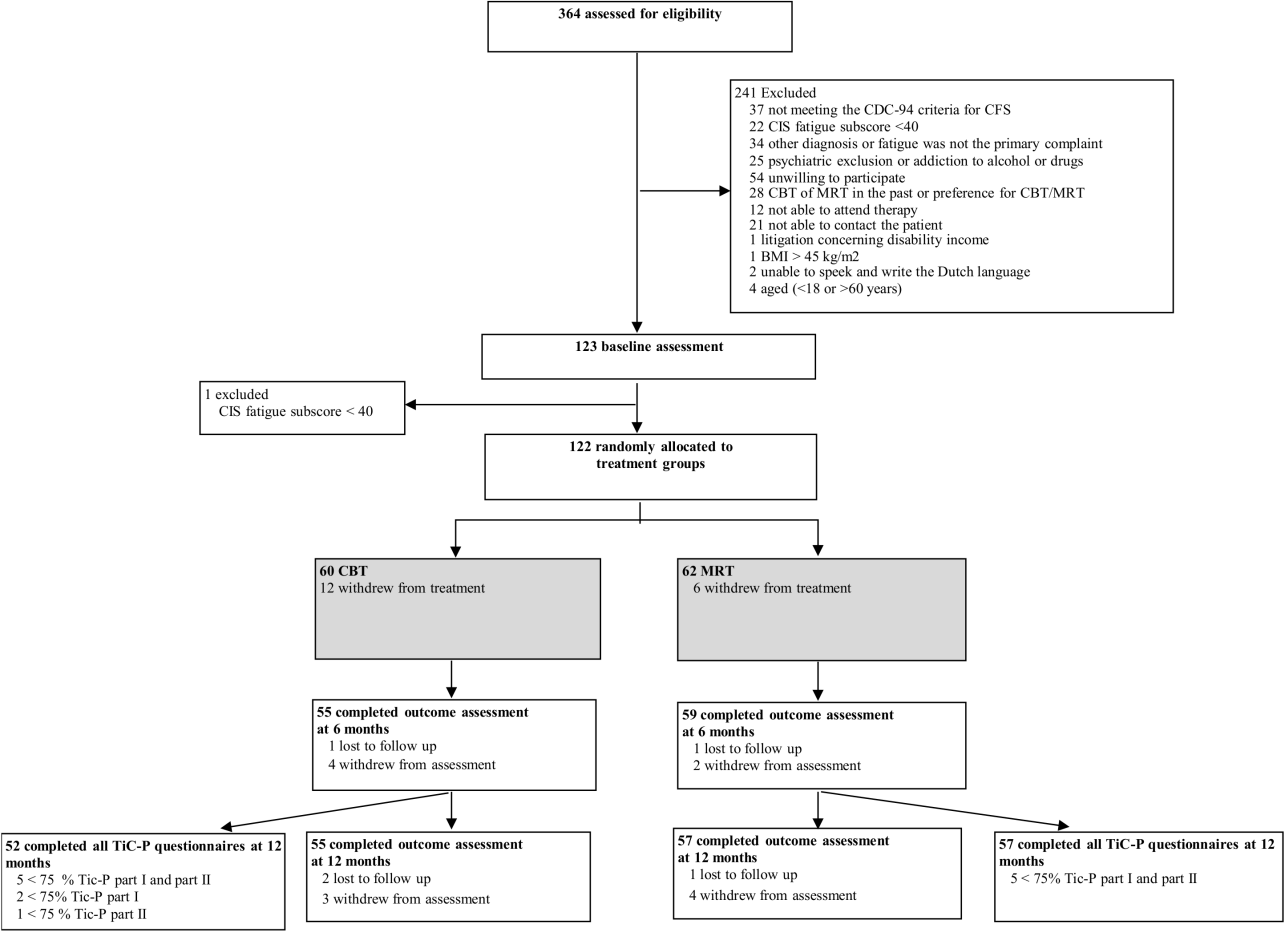
CONSORT flow diagram. CFS = chronic fatigue syndrome, CIS = Checklist Individual Strength, MRT = Multidisciplinary rehabilitation treatment, CBT = Cognitive behavioural therapy, BMI = Body Mass Index, TiC-P = Trimbos/iMTA questionnaire for Costs associated with Psychiatric Illness.

122 participants were included in the trial and completed the baseline assessment (Fig 1). Of these 122, 62 participants were randomised to MRT and 60 to CBT. The treatment effects of the 122 patients included have been described previously in the study of Vos-Vromans et al. [14]. Less than 75% of the questionnaires of the TiC-P were available from 13 patients (5 MRT and 8 CBT) and were therefore excluded from further analysis (Fig 1). 109 patients (57 MRT and 52 CBT) remained in the analysis. Of 1,744 questionnaires, 17 were incomplete or missing and were imputed (4 TiC-P part I, 13 TiC-P part II) using the above-mentioned method of imputation. Patient characteristics at baseline in the MRT group did not significantly differ from those in the CBT group. Table 2 shows the baseline characteristics of all participants stratified according to their intervention group.

**Table 2:** Baseline characteristics (N = 109).

Variable	MRT (N = 57)	CBT (N = 52)
Age, mean	40.4 (10.3)	41.6 (12.1)
Female sex, no (%)	45 (79)	43 (83)
Paid job, no (%)^**a**^	39 (68.4)	27 (51.9)
Weekly hours in paid job, mean ^**a**^	26.1 (11.2)	29.8 (10.2)
Living situation ^**b**^:		
Living alone, no (%)	14 (24.6)	10 (19.2)
Married or living together	36 (63.2)	33 (63.5)
Living apart together	1 (1.8)	5 (9.6)
Living with parents	5 (8.8)	4 (7.7)

Data are mean (SD) unless otherwise stated.

MRT = Multidisciplinary rehabilitation treatment, CBT = Cognitive behavioural therapy, CIS = Checklist Individual Strength, EQ-5D-3L = EuroQol-5Dimensions, SF-36 = Short-Form 36, PCS = Physical component summary score, MCS = Mental component summary score.

^a^ Assessed with the TiC-P in the first month after baseline.

^b^ Assessed at referral.

### Differences in effects between MRT versus CBT

Table 3 shows the treatment effect of MRT versus CBT. The estimated effect of MRT in comparison with CBT is -6.48 (95% CI: -11.54 to -1.42). At 52 weeks fatigue is significantly lower in patients from the MRT group in comparison with CBT. The estimated effect of MRT in comparison with CBT on quality of life is 3.53 (95% CI: -0.67 to 7.74) and 1.36 (95% CI: -2.28 to 5.00) for the PCS and MCS, respectively. The estimated effect for the QALY is 0.09 (95% CI: -0.02 to 0.19).

**Table 3:** Estimated effect of MRT versus CBT.

Outcome	MRT Mean (SD) (N = 57)	CBT Mean (SD) (N = 52)	Estimated effect of MRT vs CBT [95% CI] at 52 weeks^a^	Incremental effect of MRT vs CBT^b^
Fatigue				
Baseline	51.42 (5.19)	50.88 (5.36)		
26 weeks	33.12 (14.07)	36.84 (13.18)		
52 weeks	33.84 (14.33)	40.27 (12.29)	-6.48 [-11.54, -1.42]*	6.43
SF-36, PCS				
Baseline	31.02 (8.07)	32.04 (7.49)		
26 weeks	40.39 (10.43)	37.67 (10.90)		
52 weeks	40.19 (11.29)	36.61 (10.37	3.53 [-0.67, 7.74]	3.66
SF-36, MCS				
Baseline	46.40 (9.30)	44.83 (8.46)		
26 weeks	52.75 (7.08)	50.41 (8.93)		
52 weeks	51.10 (10.22)	50.25 (9.00)	1.36 [-2.28, 5.00]	0.86
QALY				
Baseline	0.48 (0.25)	0.56 (0.24)		
26 weeks	0.71 (0.17)	0.62 (0.31)		
52 weeks	0.69 (0.28)	0.61 (0.27)	0.09 [-0.02, 0.19]	0.05
QALY (UK Brazier tariff)				
Baseline	0.59 (0.07)	0.58 (0.09)	-0.003 [-0.03, 0.02]	0.00
26 weeks	0.65 (0.06)	0.63 (0.07)		
52 weeks	0.64 (0.08)	0.64 (0.06)		

MRT = Multidisciplinary rehabilitation treatment, CBT = Cognitive behavioural therapy, QALY = Quality-adjusted life-year.

^a^ Estimated effect of MRT versus CBT: Values are calculated with linear mixed models with centre, time, treatment allocation and time by treatment allocation as covariates (unstructured).

* indicates a statistically significant effect of p<0.05.

^b^ Results of the cost-effectiveness analyses: Values are calculated with 5000 bootstrap analyses in the base-case.

### Differences in costs between MRT versus CBT

The mean costs per treatment group are presented in Table 4. Healthcare costs are significantly higher for the MRT group compared to the CBT group (difference €5,681, 95% CI: €4,632 to €6,793). Patient and family costs are not significantly different between MRT and CBT (difference -€1,457, 95% CI: -€3,470 to €146). As part of the productivity costs is due to absenteeism, patients were able to fill in questions regarding loss of productivity while at work. During analyses it was noticed that it was not always clear to the patient whether or not to fill in this part of the questionnaire, making this part less reliable. Therefore loss of productivity while at work was excluded from analysis. Productivity costs due to absenteeism are not significantly different between the MRT and CBT groups (difference €1,263, 95% CI: €-667 to €3,146). The total societal costs are significantly higher for patients allocated to MRT in comparison with CBT (difference €5,389, 95% CI: €2,488 to €8,091).

**Table 4:** Mean costs (in €).

	Mean per group		Mean difference (95% CI)^a^
Cost type	MRT (N = 57)	CBT (N = 52)	
**Healthcare costs**			
General practitioner care	152.79	162.56	-10 [-62 to 48]
Mental healthcare specialist	211.25	163.55	48 [-97 to 190]
Paramedical care	255.37	182.31	73 [-103 to 288]
Medical specialist care	125.88	108.15	18 [-52 to 93]
Hospital care	286.05	185.74	100 [-270 to 614]
Medication and OTC medication	90.13	136.56	-46 [-115 to 18]
Alternative healers	89.10	93.33	4 [-78 to 93]
Company physician	560.38	299.62	261 [37 to 479]
Interventions (MRT and CBT)	7,210.89	1,922.59	5,284 [4,568 to 5,979]
**Total**	**8,989.06**	**3,308.43**	**5,681 [4,632 to 6,793]**
**Patient and family costs**			
Travelling and parking	237.95	129.48	108 [82, 133]
Informal care	1,393.25	2,933.07	-1,606 [-3637 to -30]
**Total**	**1,635.10**	**3,021.11**	**-1,457 [-3,470 to 146]**
**Productivity costs**	**3,716.71**	**2,434.98**	**1,263 [-667 to 3,146]**
**Societal costs**	**1,4307.95**	**8,845.71**	**5,389 [2,488 to 8,091]**

MRT = Multidisciplinary rehabilitation treatment; CBT = Cognitive behaviour therapy; OTC = Over the counter medication.

^a^ The upper and lower confidence limits at the 2.5^th^ and 97.56^th^ percentile based on 1000 bootstrap replications.

### Cost-effectiveness and cost-utility from a societal perspective

**Fig 2:**
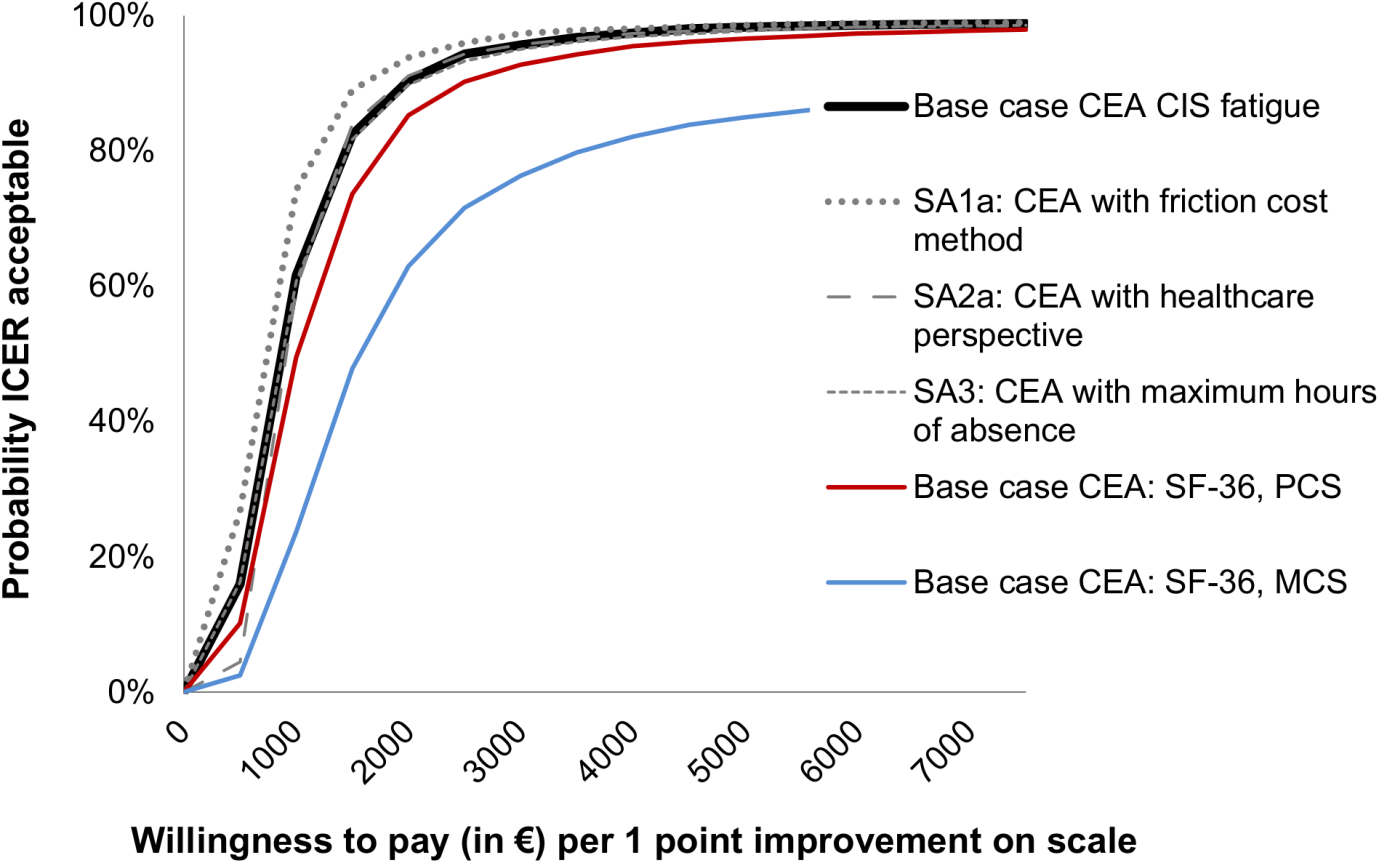
Acceptability curves of the cost-effectiveness (outcome CIS fatigue and SF-36) at 52 weeks follow-up. SA = Sensitivity analysis, CEA = Cost-effectiveness analysis, CIS = Checklist Individual Strength, SF-36 = Short-form 36, PCS = Physical component summary score, MCS = Mental component summary score.

**Fig 3:**
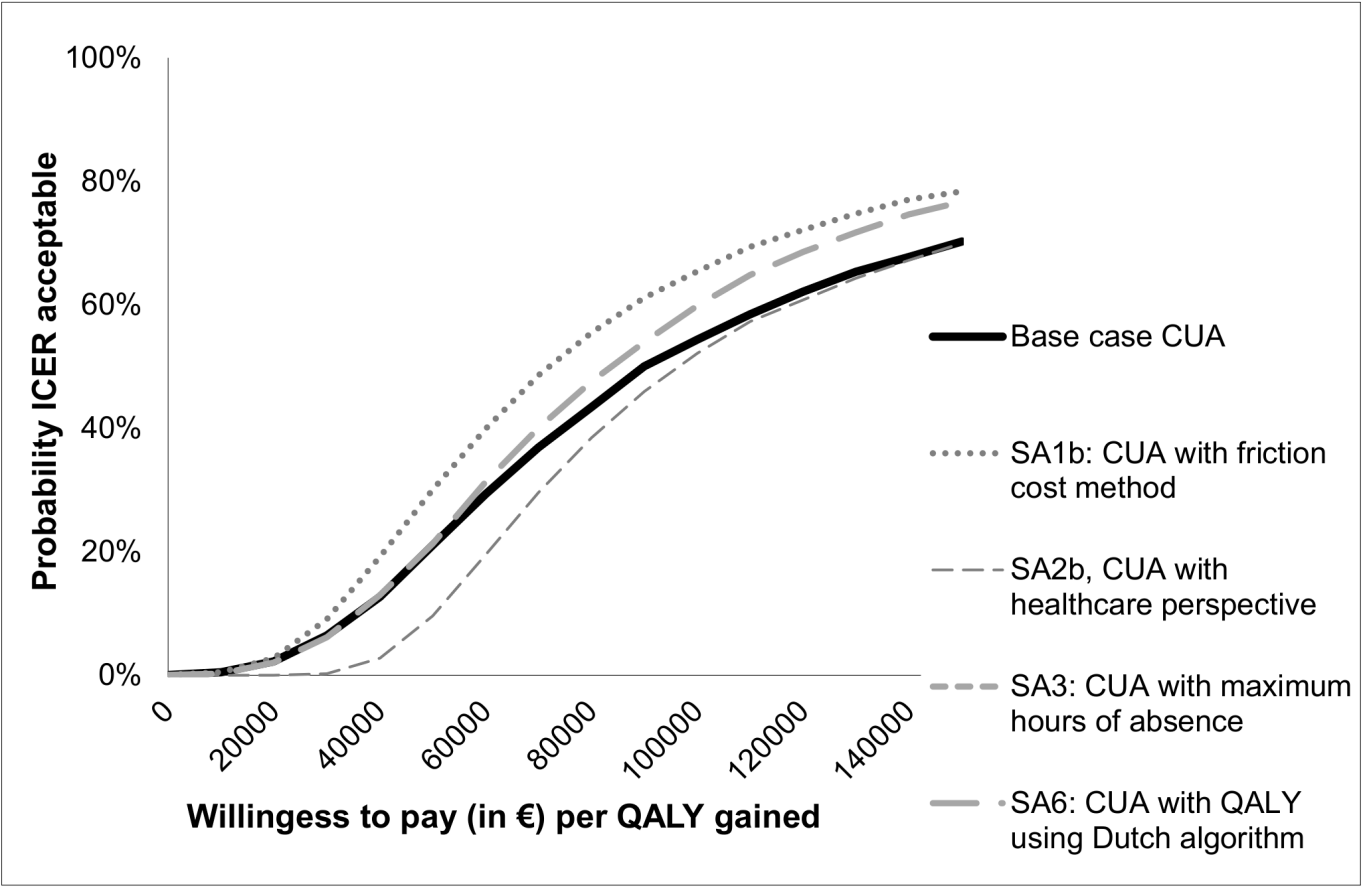
Acceptability curves of cost-utility (QALY) at 52 weeks follow-up. SA = Sensitivity analysis, CUA = Cost-utility analysis, QALY = Quality-adjusted life-year.

**Fig 4:**
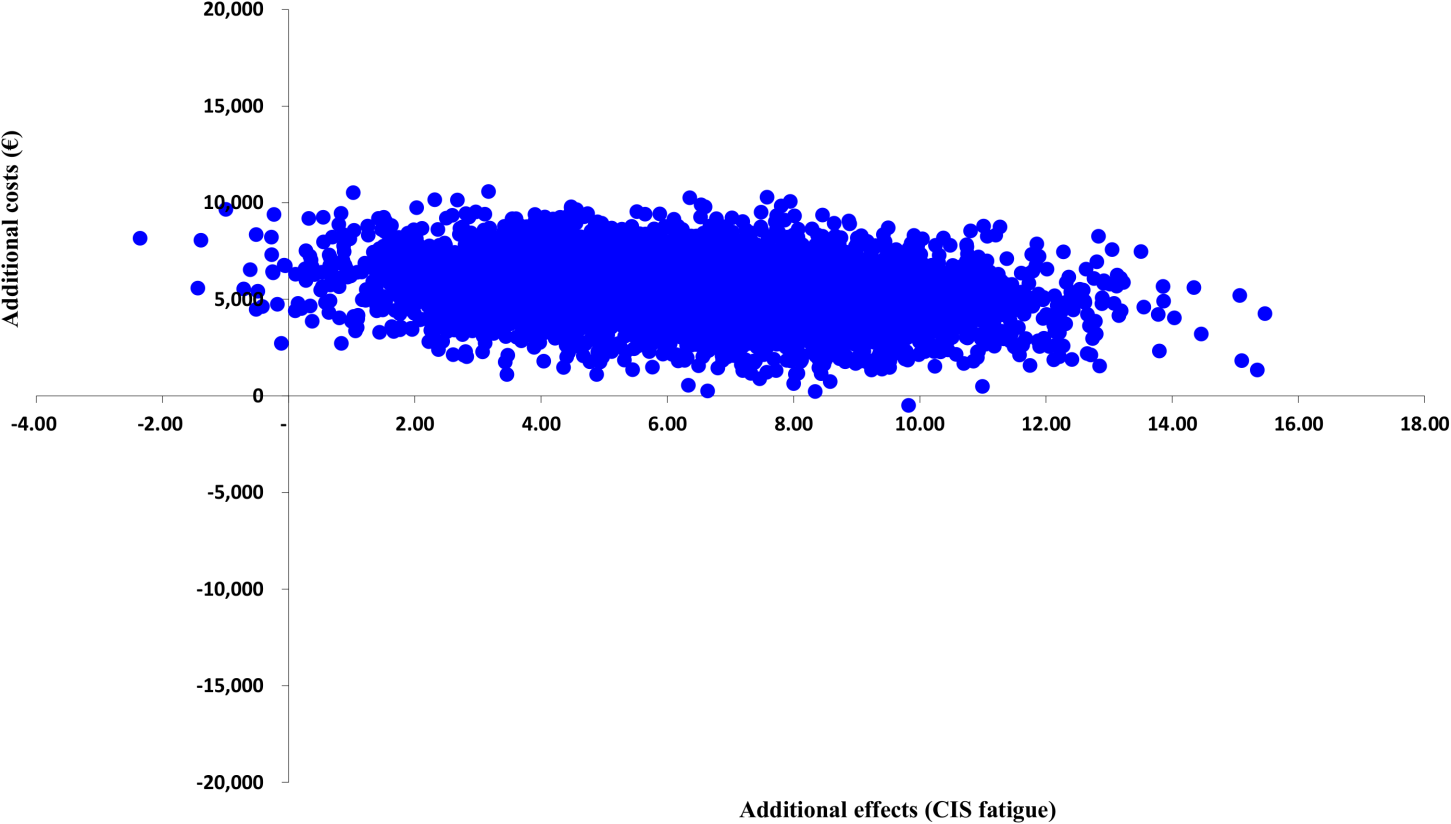
Cost-effectiveness plane (primary outcome: CIS fatigue). CIS = Checklist Individual Strength.

**Fig 5:**
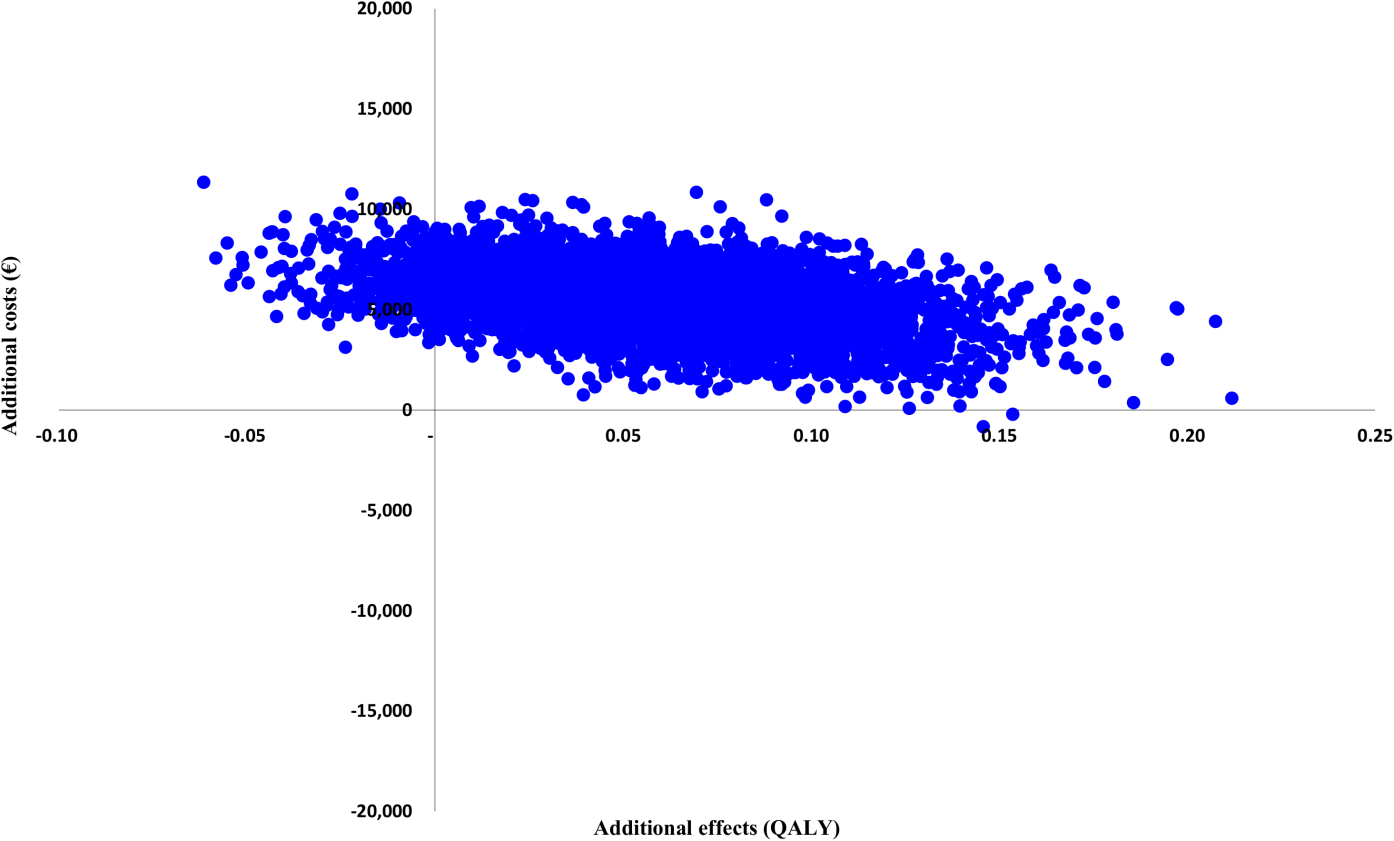
Cost-utility plane (primary outcome: QALY). QALY = Quality-adjusted life-year.

Figs 2 and 3 present the cost-effectiveness acceptability curves of the base-case cost-effectiveness and cost-utility analysis. In the cost-effectiveness analysis, the slope of the MRT acceptability curves for fatigue and quality of life are steep and increase instantly to a 94–99% likelihood of being a more cost-effective option from a societal perspective. The ICER is €856 per unit of the CIS fatigue subscale, meaning that the costs for improvement on the CIS fatigue subscale are low. For the SF-36 PCS and MCS the ICER is €1,505 and €6,416, respectively. In the cost-utility analysis, considering a threshold of €27,000 per QALY, MRT has a 5% likelihood of being more cost-effective from a societal perspective. When changing the threshold to €60,000, MRT has a 29% likelihood of being more cost-effective. The ICER is €11,8074 per QALY, meaning that the costs per QALY gained are high. Table 5 shows the cost-effectiveness and cost-utility results with the different ICER values. Cost-effectiveness and cost-utility planes are shown in Figs 4 and 5. The x axis represents the incremental level of effectiveness of the outcome and the y axis represents the additional total costs. Both figures show a higher additional effect and higher additional total costs of MRT versus CBT.

**Table 5:** Mean total costs and group differences at 52 weeks after baseline (N = 109).

Analysis		Outcomes			Costs (€)			ICER
			MRT	CBT	MRT	CBT	Mean cost-differences (95% CI) ^a^	(€/unit of outcome)^b^
	Base	CIS	30.16	23.73	14,308	8,846	5,389 [2,488 to 8091]	856
	Base	SF-36, PCS	40.23	36.57	14,308	8,846	5,389 [2,488 to 8,091]	1,505
	Base	SF-36, MCS	51.27	50.27	14,308	8,846	5,389 [2,488 to 8,091]	6,416
	Base	QALY	0.65	0.60	14,308	8,846	5,389 [2,488 to 8,091]	118,074
	Sens 1a	CIS	30.16	23.73	11,117	6,588	4,450 [2,100 to 6,518]	682
	Sens 1b	QALY	0.65	0.60	11,117	6,588	4,450 [2,100 to 6,518]	94,018
	Sens 2a	CIS	30.16	23.73	8,989	3,308	5,681 [4,632 to 6,793]	903
	Sens 2b	QALY	0.65	0.60	8,989	3,308	5,681 [4,632 to 6,793]	124,519
	Sens 3a	CIS	30.16	23.73	15,133	9,558	5,541 [2,062 to 8,940]	861
	Sens 3b	QALY	0.65	0.60	15,133	9,558	5,541 [2,062 to 8,940]	118,749
	Sens 4	EET4	0.80	0.62	14,308	8,846	5,389 [2,488 to 8,091]	29,970
	Sens 5	CIS improved	0.25	0.49	14,308	8,846	5,389 [2,488 to 8,091]	22,807
	Sens 6	QALY	0.71	0.67	14,308	8,846	5,389 [2,488 to 8,091]	109,310

ICER = Incremental cost-effectiveness ratio, MRT = Multidisciplinary rehabilitation treatment, CBT = Cognitive behavioural therapy, Base = Base-case analysis, Sens = Sensitivity analysis, CIS = Checklist Individual Strength subscale fatigue, SF-36 = Short-Form 36, CIS Improved = outcome parameter used for improvement, patients are improved if the CIS fatigue subscale was <35, EET4 = Improvement and satisfaction questionnaire, question 4 “Is there a difference in your daily activities now compared to your situation before treatment started? (‘1’ = improved and ‘0’ = not improved), QALY = Quality-adjusted life-years.

^a^ The upper and lower confidence limits are the 2.5^th^ and 97.5^th^ percentile based on 1000 bootstrap replications.

^b^ The upper and lower confidence limits are the 2.5^th^ and 97. 5^th^ percentile based on 5000 bootstrap replications.

### Sensitivity analyses

**Fig 6:**
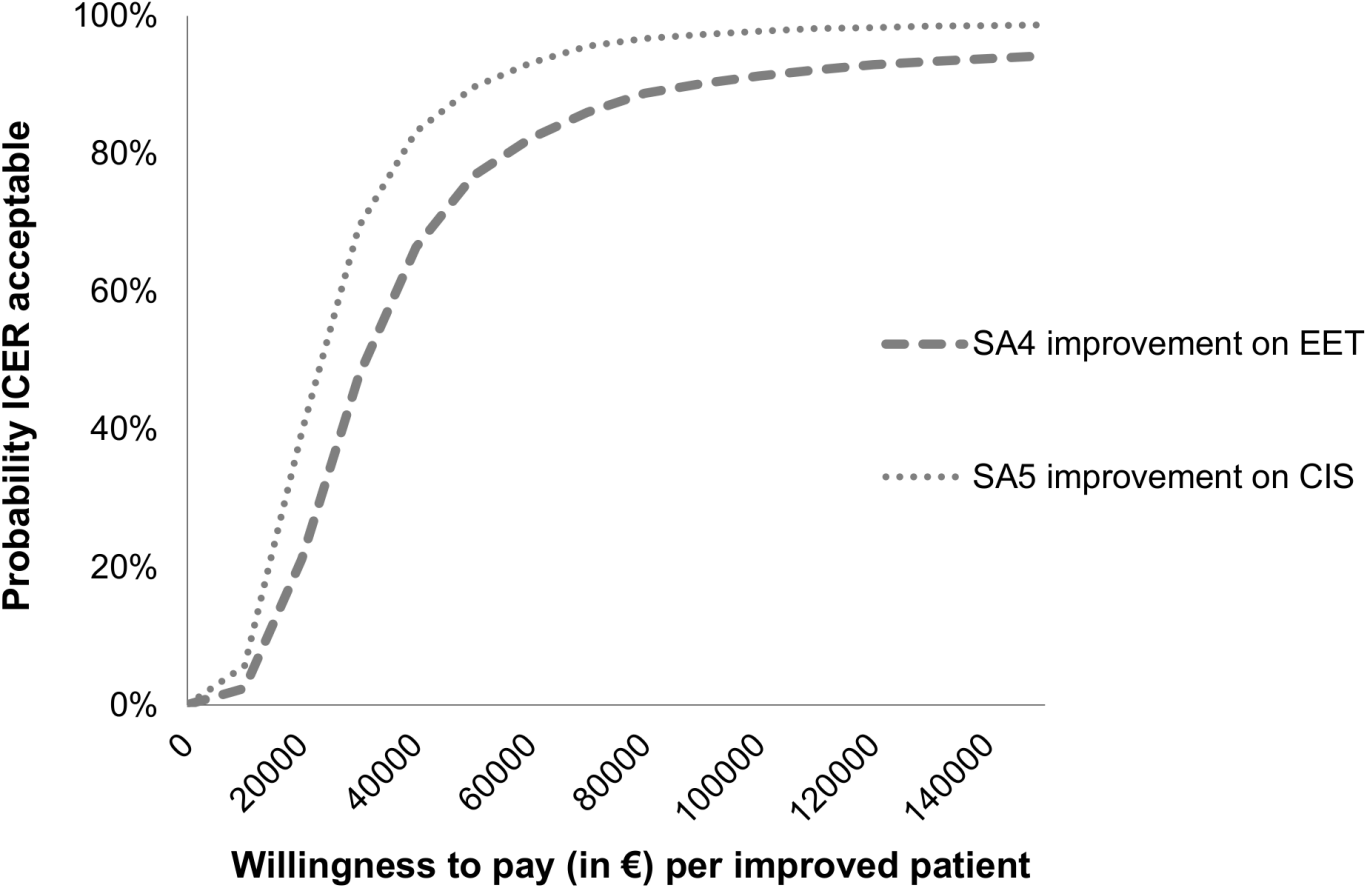
Acceptability curves (sensitivity analysis) of cost-effectiveness (outcome improvement on CIS and EET) at 52 weeks follow-up. SA4 = Sensitivity analysis 4, EET = Improvement and satisfaction questionnaire, question 4 “Is there a difference in your daily activities now, CIS = Checklist Individual Strength.

Table 5 shows the mean costs per treatment group for the different costs and effect scenarios used in the base-case and sensitivity analyses. In all scenarios, MRT has the highest mean costs. Only small differences exist between the different methods of costing, except for the friction method. Using the friction method for costing productivity losses, the costs in both treatment groups decreased: 26% in MRT and 22% in CBT, respectively. Varying the cost or QALY outcome parameters revealed similar results for the base-case and sensitivity analyses. In the utility analysis CBT is still the most favourable treatment and in the cost-effectiveness analysis the MRT is the most favourable treatment. As shown in Figs 2 and 3, the sensitivity analyses did not have a large impact on the results of the cost-utility acceptability curves. When looking at the different scales for improvement (sensitivity analyses 4 and 5) (Fig 6), the probability of MRT being more cost-effective is higher in comparison with CBT. Since we do not know exactly how much society is willing to pay for an improved patient, the probability of MRT being cost-effective is unknown.

## Discussion

As a part of the RCT studying the difference in effectiveness of MRT compared to CBT, an economic evaluation was executed to assess the difference in cost-effectiveness and cost-utility between MRT and CBT. To our knowledge, an economic study comparing CBT and MRT has never been done. Due to limited resources and the high demands on healthcare, economic evaluations have an important role in decisionmaking and health policy. Policy makers need to make decisions on how to optimize the allocation of available resources; accordingly, this study is important for policy makers as well as for patients, physicians and therapists.

MRT is more likely to be cost-effective in regard to the disease-specific outcomes fatigue severity and the physical and mental components of health-related quality of life. CBT is more likely to be cost-effective when QALYs are the outcome of interest.

### Cost-effectiveness and cost-utility

Cost-effectiveness was analysed from a societal perspective over a period of 52 weeks after baseline. Societal costs, which were mainly dominated by the costs of the intervention, were significantly higher in the MRT group in comparison with the CBT group. While MRT was associated with statistically significant improvements in disease-specific health status, this was not reflected in generic health status. The incremental effect of MRT was higher for fatigue and quality of life measured by the SF-36 and lower for the QALY. This led to contradictory results in the cost-effective and cost-utility analyses. In all cost-effectiveness base-case and sensitivity analyses, MRT was the most efficient strategy for treating patients with CFS. In all cost-utility analyses, CBT turned out to be the most cost-efficient strategy for treatment. The latter results were also found in studies comparing CBT with other treatments or with a natural course group [10,11,36]. Due to higher costs for the MRT intervention together with similar effects on the QALY, the incremental costs for a QALY are much higher for MRT in comparison with CBT. The results of the cost-effectiveness analysis are different from the results of the cost-utility analysis. The question arises whether our findings reflect an absence of a clinically significant treatment effect or, alternatively, a lack of sensitivity of the generic quality of life measures to detect a clinically meaningful improvement in patients with CFS. Differences between generic health-related quality of life measures and disease-specific measures have been discussed in previous research [37–39]. In patients who are chronically ill, treatments like MRT often focus on improving autonomy and the patient’s participation in society. Neither of these domains is not included in the EQ-5D-3L. Future studies should assess the extent to which differences in the EQ-5D-3L following treatment reconcile with improvements in disease-specific measurements for patients with CFS after treatment. In addition, as van Leeuwen et al. stated in their study in 2015 [40], the Adult Social Care Outcomes Toolkit and the ICEpop CAPability might also be valuable outcome measures in economic evaluations of care interventions because they are at least as reliable as the EQ-5D-3L and are associated with aspects of quality of life broader than health, for example occupation, dignity, control over daily life and the ability to ‘do’ and ‘be’ the things that are important in life [40].

Looking at the cost-effectiveness of treatment and considering improvement as the main outcome measure, cost-effectiveness increases in comparison with using the QALY in these analyses. But these criteria for improvement are based mainly on statistical methods and not on the patient’s own opinion. For further research, it is recommended that improvement be evaluated based on domains that are important for the individual patient and also to obtain insight into what society is willing to pay for an improved patient with CFS in order to facilitate cost-effectiveness analysis of treatments. During the process of deciding how resources are to be allocated, it is important to take into account both disease-specific and generic health status measurements, as an underestimation of the treatment effect might occur using only generic measurements.

### Implementation of treatment

Based on the differences between the results of the cost-utility and cost-effectiveness analyses, discussion will occur regarding whether MRT should be implemented in other rehabilitation centres. The results of a multi-centre RCT [14] showed that, quality of life increases and fatigue decreases in both CBT and MRT. But over time MRT is more effective in decreasing fatigue compared with CBT. During the decisionmaking process regarding which treatment a patient will receive, this long-term effect is probably more important to the patient and the clinician than the fact that one of the two treatments has lower societal costs. Increasing the cost-effectiveness is important for clinicians and patients. Regarding this, it might be worthwhile to take other components into account when deciding to implement MRT as a potential treatment for patients with CFS in the Netherlands. The scarcity of effective treatments for patients with CFS and patient preferences are relevant issues when making healthcare decisions. At this moment there is a scarcity of effective treatments for patients with CFS [41], which might stimulate the implementation of MRT. Preferences of the patient should also be taken into account when making a healthcare decision. In this trial significantly more patients from the MRT group would recommend the treatment to others in comparison with patients from the CBT group [14], which might give an impression of the preferences of treatment for patients with CFS. Further research is needed on this topic. Another point which should be taken into account is the fact that costs for the MRT were probably overestimated since treatment was new. Costs for MRT might decrease as this treatment is executed more routinely and therapists are better skilled at treating patients more effectively. In a post-hoc analysis the first patients in the MRT group had significantly more contact hours compared to the patients who were included later in the trial. In CBT such development was not found. Additionally, costs of both interventions might become lower when therapists are able to decrease the number of sessions needed to achieve a patient’s personal goal or when specific interventions are not needed to achieve a patient’s goal. In clinical practice this is already the case, but due to the treatment protocols in this trial, in which a minimum number of hours of treatment were prescribed, this was not an option.

### Future research

In the MRT and CBT interventions the societal costs are dominated by the costs for the intervention. Future research is needed to study how the interventions can be more cost-effective. It might be possible to offer parts of these interventions in groups of patients instead of to individuals, which will decrease costs of treatment. Another option to decrease the costs of MRT is to offer the patient fewer interventions or the same interventions less frequently, or to provide interventions in web-based programmes [42]. Whether the effect of treatment remains similar should be evaluated in future studies. Since productivity costs are highest in the MRT group, it is also worthwhile paying more attention to returning to work. In CBT returning to work is part of the protocol. In MRT returning to work is included in the treatment only if it is part of the patient’s personal goal. If not, returning to work is not part of the MRT procedure. In order to decrease the costs from a societal perspective, making ‘returning to work’ a fixed part of the MRT protocol is useful, despite the fact that it might not be a personal goal of the patient. Whether this changes the cost-effectiveness of treatment needs to be evaluated in future research.

### Strengths and limitations

This trial has a number of strengths; in particular, internal validity is high due to randomization and concealed allocation procedures, a priori trial registration, intention to treat analysis and pre-specification of primary outcomes and analytic methods. However, data used in our analyses were derived from a single RCT, presenting a potential limitation to the generalizability of our findings. Furthermore, as only patients who were specifically referred for a treatment in secondary care were included, makes the results highly generalizable to daily care of patients with CFS. On the other hand, as 37 patients did not meet the CDC-94 criteria of CFS and were excluded before randomization, 54 patients were unwilling to participate in the trial, and the data of only 109 patient was used in the analysis should be taken into account when generalizing the results to the total population of patients with fatigue as a main complaint who are referred to secondary healthcare. In clinical practice CDC-94 criteria are not always used in deciding to treat patients in a rehabilitation centre. Furthermore, some patients were unwilling to participate in the trial, which can be seen as selection bias.

Other strengths are that we were able to recruit the intended number of patients based on the power analyses and the overall dropout rate was rather low, and several sensitivity analyses proved that the results are rather robust. Furthermore, all care-providers were well trained and overall the participating treatments centres showed similar treatment results.

However, there are some other limitations: First, we relied on self-reported information regarding healthcare, productivity losses and patient-family costs. There may be issues of accuracy with this approach, but it was largely unavoidable, given the impossibility of registering otherwise. Moreover, as these measurements were similar in both groups, these issues did not affect comparability. Other studies have shown that the method used is acceptable [43,44]. Second, the loss of productivity while being at work was not taken into account. Future studies should include loss of productivity while at work by using the TiC-P with an extra instruction to the patient on how and when to fill in this part of the questionnaire. Third, for some patients we had to impute data of the TiC-P, which can have influenced our results. Finally, the costs and effects were measured only during a period of 52 weeks. It is possible the costs and effects change after 52 weeks. This might lead to other conclusions regarding cost-effectiveness. For future research it would be interesting to perform the study over a longer period after ending the interventions.

### Clinical implication

Although MRT is at long term more effective in reducing fatigue compared with CBT, the cost-effectiveness and cost-utility show mixed results making it difficult to provide clinical implications. More research is needed. It is a challenge for clinicians to improve and evaluate study how the interventions resulting in a less costly but at least equally effective treatment in order to improve the cost-effectiveness of both treatments.

## Conclusion

Using fatigue severity as primary outcome for cost-effectiveness, MRT is more likely to be cost-effective in comparison with CBT. Considering the QALY, a generic quality of life measure, CBT is more cost-effective than MRT. To further improve the interpretation of cost-effectiveness analysis of treatments in patients with CFS it is important to clearly define criteria for improvement and how much money society is willing to pay for an improved patient.

## Supporting information

S1 File Design trial. (PDF)

S2 File CONSORT checklist. (PDF)

S3 File CHEERS checklist. (PDF)
