# Supplementary material for: Economic evaluation of multidisciplinary rehabilitation treatment versus cognitive behavioural therapy for patients with chronic fatigue syndrome: A randomized controlled trial
Source: PLoS One. 2017 Jun 2;12(6):e0177260. doi: 10.1371/journal.pone.0177260 (PMC5456034; doi:10.1371/journal.pone.0177260)
Supplement: S2 File — (PDF) [file pone.0177260.s002.pdf]

# CHEERS checklist

## *Economic evaluation of multidisciplinary rehabilitation versus cognitive behaviour therapy for patients with chronic fatigue syndrome: a randomized controlled trial (PONE-D-16-40930R1)*

| **Section/item** | **Item no** | **Reported on Page No/** |
|---|---|---|
| **Title and abstract** | | |
| Title | 1 | 1 |
| Abstract | 2 | 3-4 |
| **Introduction** | | |
| Background and objectives | 3 | 5-7 |
| **Methods** | | |
| Target population and subgroups | 4 | 7-8 |
| Setting and location | 5 | 7-8 |
| Study perspective | 6 | 7-8 |
| Comparators | 7 | 8 |
| Time horizon | 8 | 9 |
| Discount rate | 9 | 9-11 |
| Choice of health outcomes | 10 | 8-9 |
| Measurement of effectiveness | 11 | 8-9 |
| Measurement and valuation of preference based outcomes | 12 | 9-13 |
| Estimating resources and costs | 13 | 9-13 |
| Currency, price date, and conversion | 14 | 10 |
| Choice of model | 15 | 13-15 |
| Assumptions | 16 | 16 |
| Analytical methods | 17 | 13-16 |
| **Results** | | |
| Study parameters | 18 | 17-22 |
| Incremental costs and outcomes | 19 | 18-21 |
| Characterising uncertainty | 20 | 22-23 |
| Characterising heterogeneity | 21 | Not applicable |
| **Discussion** | | |
| Study findings, limitations, generalisability, and current knowledge | 22 | 24-30 |
| **Other** | | |
| Source of funding | 23 | 30 |
| Conflicts of interest | 24 | 31 |
